# Adapting conversational strategies in information-giving human-agent interaction

**DOI:** 10.3389/frai.2022.1029340

**Published:** 2022-10-25

**Authors:** Lucie Galland, Catherine Pelachaud, Florian Pecune

**Affiliations:** ^1^Département d'Informatique de l'ENS, ENS, CNRS, PSL University, Paris, France; ^2^ISIR, CNRS, Paris, France; ^3^Department of Computer Science, Waseda University, Tokyo, Japan

**Keywords:** interaction human-agent, dialog manager, reinforcement learning, adaptation, task and social dialog acts

## Abstract

In this work, we focus on human-agent interaction where the role of the socially interactive agent is to optimize the amount of information to give to a user. In particular, we developed a dialog manager able to adapt the agent's conversational strategies to the preferences of the user it is interacting with to maximize the user's engagement during the interaction. For this purpose, we train an agent in interaction with a user using the reinforcement learning approach. The engagement of the user is measured using their non-verbal behaviors and turn-taking status. This measured engagement is used in the reward function, which balances the task of the agent (giving information) and its social goal (maintaining the user highly engaged). Agent's dialog acts may have different impact on the user's engagement depending on several factors, such as their personality, interest in the discussion topic, and attitude toward the agent. A subjective study was conducted with 120 participants to measure how third-party observers can perceive the adaptation of our dialog model. The results show that adapting the agent's conversational strategies has an influence on the participants' perception.

## 1. Introduction

Socially intelligent agents (SIA) (Cassell, 2001) are virtual entities with a human-like appearance. They communicate verbally as well as non-verbally and are used in human-machine interactions to play different roles, such as assistant, tutor, guide, or companion (Lugrin and Rehm, 2021). To communicate efficiently, these agents should be able to adjust in real-time to their users' engagement, one of the features that ensure high quality users' experience (O'Brien and Toms, 2008). Engagement can be defined as “the value that a participant in an interaction attributes to the goal of being together with the other participant(s) and continuing the interaction” (Poggi, 2007), and is associated with high-level behaviors such as synchrony, alignment, mimicry, feedback, backchannel, and collaboration. These high-level behaviors can be expressed with various lower-level behaviors, such as head nods (Allwood and Cerrato, 2003), smiles (Castellano et al., 2009), mutual gaze (Nakano and Ishii, 2010), or body posture (Sanghvi et al., 2011).

In information-giving context, studies have demonstrated that addressees' level of engagement has a significant impact on their motivation, effort, and memorizing gains (Wentzel, 1999). The more people are engaged during an interaction, the more information they will remember at the end. Hence, one of the main challenges for an information-giving SIA is to dynamically manage the conversation by selecting the best dialog policy to fulfill its task (e.g., by delivering relevant information) and to maintain user's engagement at the same time (e.g., by disclosing personal information, telling jokes, or using small talk).

Manually authoring optimal dialog policies for deep and complex scenarios can be overwhelming (Rich and Sidner, 2012), and dialog authoring becomes even harder when the SIA has to adapt its behavior according to the conversational preferences of the user. Indeed, while some people will appreciate a friendly interlocutor that livens up the interaction with jokes, personal anecdotes, or opinions, others would prefer to interact with someone who solely focuses on the task (Tracy and Coupland, 1990). That is, when it gets the speaking turn, the agent should find the right balance and timing for choosing between using a task-oriented dialog act or a socially-oriented dialog act.

One solution to teach conversational agents the optimal sequence of actions to perform is Reinforcement Learning (RL). RL agents can not only learn the optimal policy to help users achieve their task, but they can also learn how to adapt their behavior to engage different types of users depending on their preferences (Walker, 2000; Li et al., 2016). Such RL approaches require a large amount of data to explore all possible dialog options before learning the optimal dialog policy. User simulators (Schatzmann et al., 2007) were created as a solution to overcome this shortcoming by approximating the behavior of real users during an interaction. User simulators have been developed to explicitly approximate different types of user, for instance, from different age categories (Georgila et al., 2010) or with different conversational preferences (Jain et al., 2018). We rely on a similar approach to simulate different types of user that prefer specific levels of social and task behavior from the agent with which they are interacting.

In this work, we present a conversational agent capable of maximizing users' engagement during an interaction while delivering specific information. More specifically, we train our agent using deep reinforcement learning to optimize its conversational strategies based on users' preferred behavior from the agent in terms of task and social behavior. We present the related works in Section 2, before introducing our conversational agent architecture in Section 3 and the NoXi corpus (Cafaro et al., 2017) in Section 4. We describe the user simulator we built in Section 5, and the agent we trained using RL in Section 6. Finally, we evaluate our agent and discuss the results in Section 7.

## 2. Related works

Using RL to optimize SIAs' behaviors during an interaction has been a topic of interest in the past years.

Some agents rely on RL to learn a new optimal policy each time they interact with a user. These agents only have a limited number of conversational turns to learn which optimal behavior to express, hence the limited number of actions at their disposal. In Leite et al. (2011), a chess companion has to choose from four different empathetic strategies to encourage a child playing chess. The robot first derives children's emotional state from their non-verbal behavior and the status of the game. This emotional state is used as a reward each turn to teach the robot which empathetic strategy is the most efficient to encourage the children. An evaluation shows that the empathetic version of the chess companion was perceived as more engaging, helpful, and also obtained higher ratings in terms of self-validation than the non-empathetic one. The robot described in Weber et al. (2018) relies on RL to improve user amusement by selecting a joke, a grimace, or a sound. The authors in Gordon et al. (2016) enhance the behaviors of a robot tutor by adding affective feedback on top of predefined task-related responses. Similarly to Leite et al. (2011), the robot tutor detects the affective state of users based on their non-verbal behaviors, and uses this emotional state as a reward to optimize its affective policy. In Biancardi et al. (2019a) and Biancardi et al. (2019b), the agent learns to optimize the first impression of users by adapting its linguistic and non-verbal style in an online manner. At each agent's speaking turn, a Q-learning algorithm selects one out of four conversational styles (Biancardi et al., 2019a) or chooses between verbal and non-verbal cues (Biancardi et al., 2019b) to improve users' first impression of the agent.

These agents focus solely on the social aspect of the interaction, either by adding a social layer on top of a predetermined scenario or by selecting appropriate social feedback. They rely on RL to select the best social option amongst a limited space, pay little if no attention to the task goal of the user, and the social option selected does not have any impact on the course of the interaction.

Some agents have been designed to address this problem by combining task and social rewards. These agents rely on RL to learn which optimal sequence of actions would help their users achieve their task and maximize their engagement at the same time, instead of relying on a pre-determined scenario.

In Shi and Yu (2018), the authors combined an emotional reward with a task reward to train an information search conversational system. They first trained a sentiment estimator that relied on dialogic, acoustic, and textual features to predict a sentiment score at the end of an interaction. Then they built a rule-based user simulator to generate synthetic interactions and train the agent. The authors found that combining both emotional and task reward resulted in shorter conversations on average. Although this work considers task and social rewards to train an agent, the actions that the system can take are all task-oriented. Another approach is taken in Yu et al. (2017), where the authors train a conversational system that interleaves task and social actions to improve the task success rate and increase user engagement. At each time step, the system chooses between a task-oriented action or a social one. The reward function used to train the system is a linear combination of four metrics: turn-level appropriateness, conversational depth, information gain, and conversation length. Their subjective evaluation showed that participants thought they were more engaged when they interacted with a system that could interleave social content within the task-oriented conversation. Overall, these works consider one single policy for their agent to optimize. None of these systems adapt their conversational behavior depending on whether their users care about the social aspect of the interaction or not, which means that the single policy learned might actually be sub-optimal. Closer to our work, the conversational recommendation agent in Pecune and Marsella (2020) learns two different dialog policies depending on its users conversational goals. Each turn, the agent selects a combination of task-oriented and socially-oriented dialog act to optimize both the task performance and the level of rapport occurring during the interaction. However, this work only focuses on dialog acts and does not consider non-verbal behavior. Hence, our research questions are:

**RQ1:** How to simulate the behavior (dialog acts and non-verbal behaviors) of users with different conversational preferences interacting with an information-giving conversational agent.
**RQ2:** How to optimize an information giving conversational agent's dialog policy to maximize both task-performance and users' engagement?

## 3. Our approach

Our aim is to build an SIA capable of delivering a certain amount of information during an interaction with a user. To convey as much information as possible and to maintain interaction quality, the agent tries to maintain user's engagement during their interaction. To this aim, the agent may rely on different conversational strategies that may involve providing details on a topic or introducing another topic (these acts are referred to as task-oriented) or that may correspond to self-disclosure, small talks, or even to telling a joke (referred to as socially-oriented). At any of its speaking turn, the conversational strategy of the agent can be either task- or socially-oriented. The agent adapts to the user's preferences in terms of topics and of balance between task/social dialog acts. By adapting dynamically the choice of its dialog acts to the user's perceived preference, the agent aims to maximize user's engagement.

**Figure 1:**
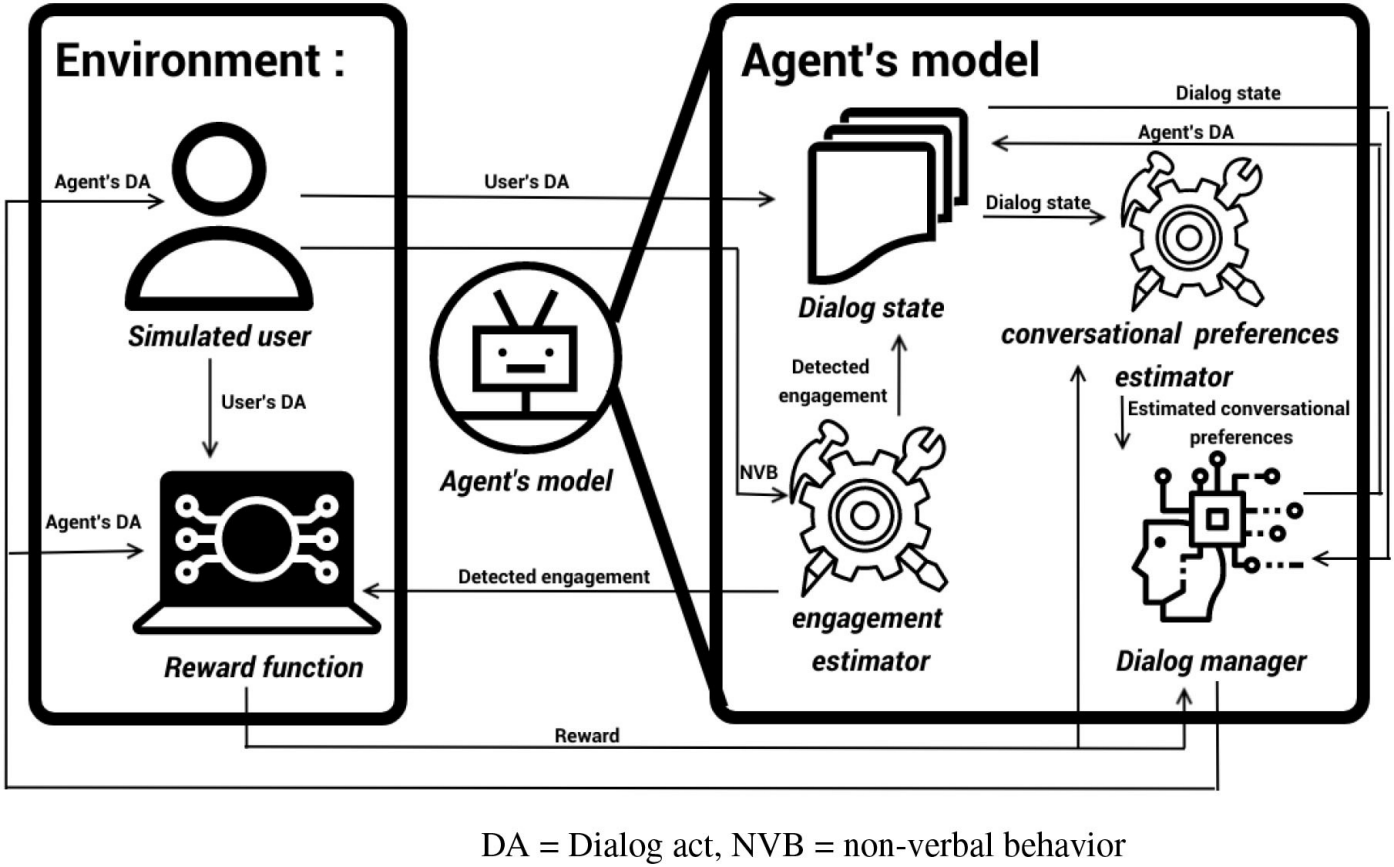
System architecture. DA, dialog act; NVB, non-verbal behavior.

To optimize the dialog policy of our SIA, we rely on Reinforcement Learning. As in Pecune and Marsella (2020), Shi and Yu (2018), and Yu et al. (2017), we build a user simulator to approximate human users' behaviors and generate enough synthetic interactions to train the agent. The use of a simulated user allows to pretrain the model and avoid the “cold start problem” where the model behaves badly in the first interactions with human users. Our agent is built in three parts that interact with each other, as represented in Figure 1: an engagement estimator, a conversation preferences estimator, and a dialog manager. This modular approach allows for more explainability of each of the modules. At each speaking turn, the simulated user module computes which intentions to convey by imitating the behavior of real users; for this, it generates a dialog act (Stolcke et al., 1998) and a sequence of non-verbal behaviors (Grimaldi and Pelachaud, 2021). Then, the engagement estimator computes the perceived level of engagement of the simulated user from the non-verbal behavior and turn-taking information, as in Sidner and Dzikovska (2002). The dialog state is then updated with this information. Depending on the simulated user's reaction to the agent's previous action, the conversational preferences estimator module updates the estimated conversational preferences of the user. The agent dialog manager then produces a dialog act based on the current dialog state and the simulated user's estimated conversational preferences. Finally, the simulated user updates its own engagement according to the agent's behavior and produces another dialog act that updates the dialog state and gives back the turn to the agent.

## 4. Corpus

To answer our first research question **RQ1** and to simulate users interacting with an information-giving conversational agent to be able to train our RL agent, we first analyze how real humans behave in a similar situation. We studied NoXi (Cafaro et al., 2017), a video corpus of dyadic interactions between experts in a specific domain (e.g., cinema, cooking, sport) and novices who wish to learn about this domain. The NoXi corpus is annotated with non-verbal behaviors (head movements, head direction, smiles, gaze direction, arm positions) for both the expert and the novice. The interactions in NoXi are also annotated with an engagement score that continuously varies between 0 (very low engagement) and 1 (very high engagement) over the course of the conversation. A Boruta analysis showed that all the annotated non-verbal behaviors were relevant for the estimation of engagement (*p* = 0.01). The most important features positively correlated with a high level of engagement are arm openness and smiles. To gain a better understanding of how novices behaved during the interaction, we automatically annotated the corpus in terms of dialog act using the DialogTag package (Malik, 2022). 0.03 % of NoXi's novice's dialog acts were annotated as questions, 96% as feedback, and 3,97% as informative statements.

**Figure 2:**
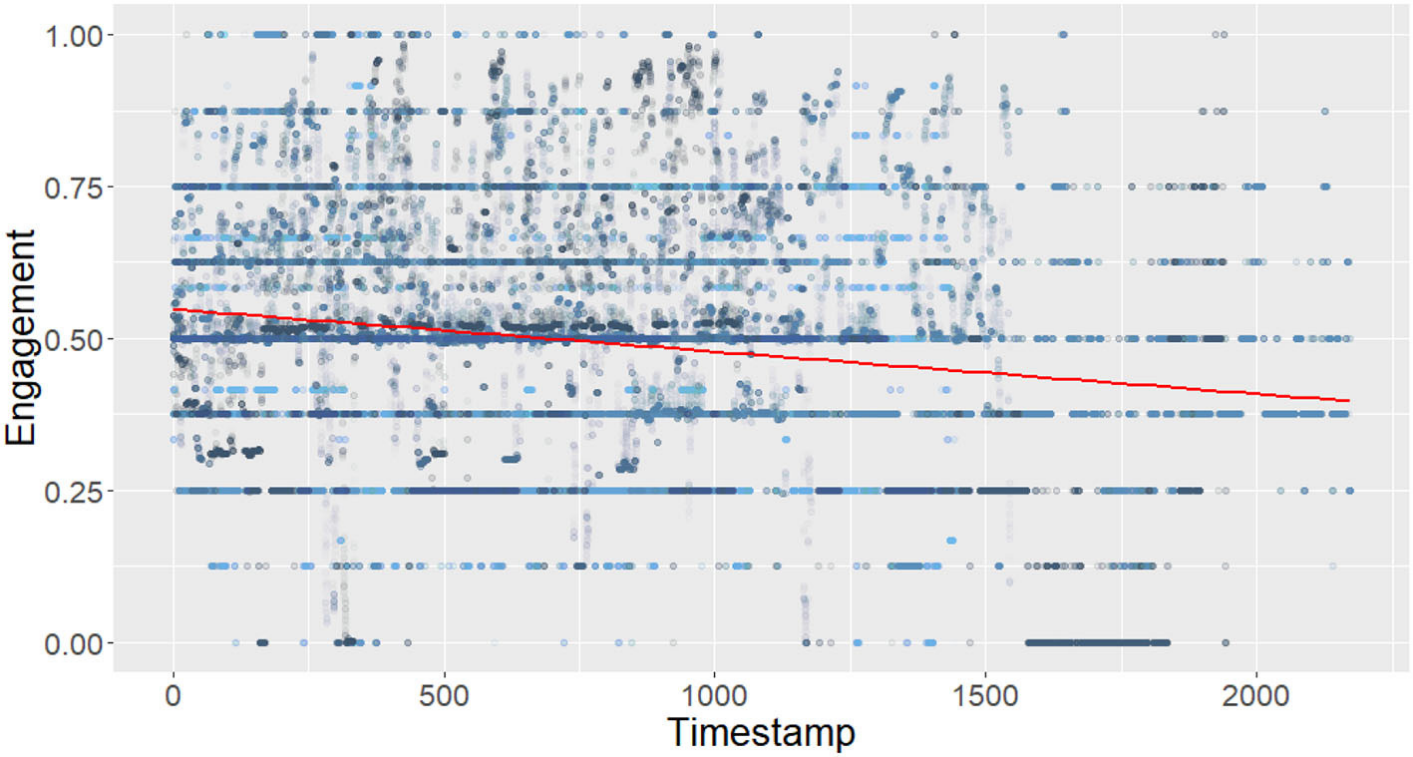
Evolution of engagement over time in NoXi.

**Figure 3:**
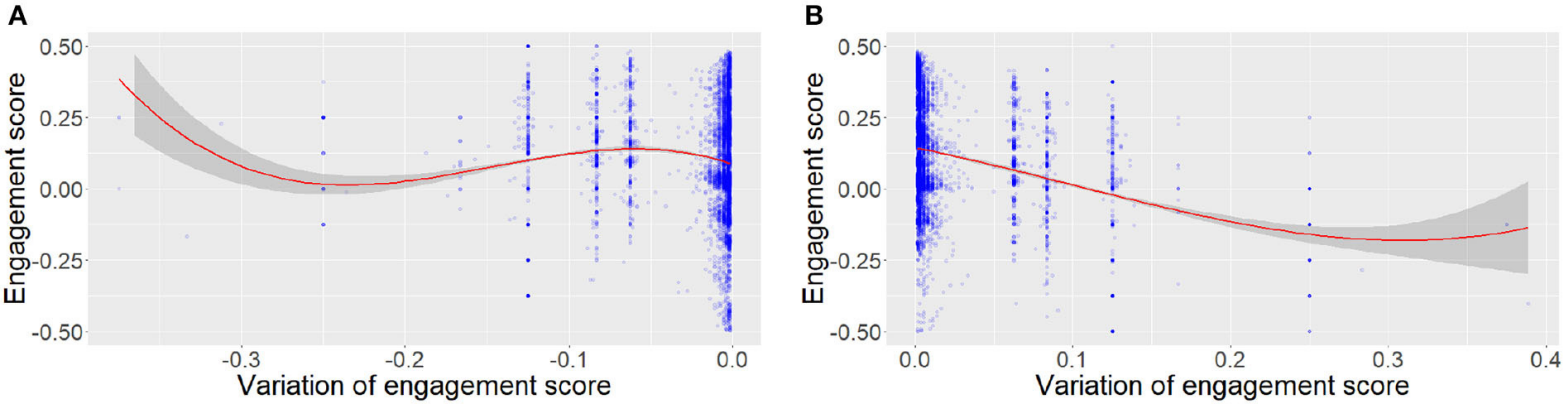
Variation of engagement in NoXi according to the current level of engagement. **(A)** Positive variations. **(B)** Negative variations.

In this paper, we specifically investigate the evolution of the engagement score during interactions. First, we analyze how engagement evolves over time. The linear regression illustrated in Figure 2 shows a significant negative effect of time on engagement (Engagement = −6.969e-05*Time + 5.480e-01 with *p* ≤ 2*e*−16 and adjusted *R*^2^ = 0.03), showing that novices slowly disengage themselves during the course of the interaction. Expending the work of Mayne and Ramsey (2001), we also analyze the relation between the variation of engagement and the actual engagement score. The linear regressions shown in Figure 3 show a significant negative effect of the absolute value of the engagement variation and the distance between the actual engagement and a neutral engagement score (for positive engagement = −0.12 * engagement variation + 0.05 with *p* ≤ 2*e*−16 adjusted *R*^2^ = 0.16) and negative [engagement = 0.38 * log(engagement variation) −4.57 with *p* ≤ 2*e*−16 and adjusted *R*^2^ = 0.001], a log is applied to negative engagement variations, as they were not normally distributed). In other words, Figure 3A shows that the current level of engagement drops further when it was previously high than when it was medium. On the other hand, Figure 3B shows that it is easier to gain engagement when the previous level of engagement was medium than when it was already high.

## 5. User simulator

To emulate the behavior of users interacting with our information-giving agent, we rely on our analyses from Section 4 to build simulated users. For each interaction, a new simulated user is created by the user simulator. Each simulated user is characterized by two features: its conversational preferences and its topic preferences.

### 5.1. User's preferences

#### 5.1.1. Conversational preferences

As shown by Pecune and Marsella (2020), people's conversational goals and preferences influence the perceived quality of an interaction. For instance, people only focusing on the task might quickly get disengaged if their interlocutor uses social conversational strategies. On the other hand, people interested in building a social relationship with their interlocutor might disengage themselves if their interlocutor only cares about the task being accomplished. In our work, we define our simulated user's conversational preferences along the following two variables:

- **(S)** ∈{0, 1}: If (*S*) = 1 the user likes to interact with an agent using social strategies, while if (*S*) = 0 the user dislikes it.
- **(T)** ∈{0, 1}: If (*T*) = 1 the user likes to interact with an agent that focuses on its task, while if (*T*) = 0 the user dislikes it.

Each of our simulated users will be assigned to one type denoting their preferences. Different types of simulated users could have one of the above preferences defined by the variables S or T or by a combination of the two:

- **(TS) user**: prefers the agent to use socially- as well as task-oriented dialog acts
- **(S) user**: prefers the agent to use socially-oriented dialog acts and will disengage when faced with task-oriented dialog acts.
- **(T) user**: prefers the agent to use task-oriented dialog acts and will disengage when faced with socially-oriented dialog acts.

#### 5.1.2. Topic preferences

The study from Glas and Pelachaud (2015) shows that people are significantly more engaged in a conversation when their interlocutor talks about a topic they like. Hence, in our work, each simulated user has preferences for certain topics. We represent the different topics to be discussed during the conversation with a matrix *SIM*. For each pair of topics (*o*_*i*_, *o*_*j*_), the value *SIM*(*o*_*i*_, *o*_*j*_)∈[0, 1] represents the similarity between the two topics. The higher the score, the closer the topics. Whenever a new simulated user is created, the matrix *SIM* is multiplied by a random vector to represent the user's topic preferences.

### 5.2. Engagement function

The engagement of the simulated user is updated at each dialog turn according to the current simulated user's state *s*∈*S*.


(1) $$\begin{array}{l} e_{t}=e_{t-1}*W\left(e_{t-1}\right)+i_{da}*l_{da}\left(e_{t-1}\right)-h_{t-1}\left(a_{t}\right)*l_{h}\left(e_{t-1}\right)+b \end{array}$$

Where:

- *e*_*t*−1_ is the previous engagement value
- *W*(*e*_*t*−1_) is a Gaussian function that represents how engagement decreases over time applied to the engagement value observed in NoXi (see Figure 3)
- *i*_*da*_ is the impact of the dialog act. It is positive if it corresponds to the user's preferences (topic and conversational), and negative otherwise.
- *h*_*t*−1_ represents the history of the conversation. A growing penalty is added to the engagement each time the agent uses a strategy to model tiresomeness faced with repetitions.
- *l*_*h*_ and *l*_*da*_ represent the non-linearity in engagement variation observed in Noxi (Figure 3).
- *b* is a Gaussian random noise that is used to model the variability of human interaction.

### 5.3. User's action choice

At each speaking turn, the simulated user outputs two types of information: a dialog act and a list of non-verbal behaviors.

#### 5.3.1. Simulated user's dialog act

As often used in the literature Schatzmann et al. (2007), the simulated user is a finite-state machine (FSM). An FSM is defined by the list of its states, its initial state, and the inputs that trigger transitions between states. For each *s*∈*S*, a function *P*(*s*):*a*→*p* gives the probability *p* of performing the action *a*. At each speaking turn given the current state *S*, an action *a* is sampled according to this distribution *P*(*s*).

At each turn, the simulated user is able to either:

- Stay silent
- Give a feedback (positive or negative): A positive feedback insists on the interest of the user namely “That is really interesting” while a negative feedback only gives acknowledgment on the agent's utterance, namely “Ok.”
- Ask for more information on a topic
- Answer if the agent asks a question
- Leave the conversation

The simulated users are built such to have similar dialog act distributions as the Noxi's novices.

#### 5.3.2. Simulated user's non-verbal behavior

The simulated user also provides non-verbal behavior to the engagement detector module. This behavior must be representative of the level of user engagement. Non-verbal behaviors are sampled from the NoXi database (Cafaro et al., 2017). At each simulated turn, the simulated user randomly selects a speaking turn in NoXi where the novice shows the same level of engagement as the simulated user. The simulated user displays the same non-verbal behaviors as the novice in the selected turn.

## 6. Agent's model

To answer to our **RQ2** and optimize our conversational agent's dialog policy to maximize both task-performance and users' engagement, we endow our agent's model with the following components: the engagement estimator estimates the engagement of the user based on their non-verbal behavior (see Section 6.1) and turn taking status, the topic manager keeps track of the user's favorite topics, the conversational preferences estimator estimates the conversational preferences of the user at each turn, and the dialog manager selects the next dialog act based on the information provided by the other modules.

### 6.1. Engagement estimator

The engagement estimator is trained using the NoXi corpus (Cafaro et al., 2017). Based on Sidner and Dzikovska (2002) and Dermouche and Pelachaud (2019) and our Boruta analysis of NoXi (see Section 4), the inputs considered are non-verbal behaviors such as arm openness, arm closed, head nod, head shake, head touch, smile, look at/away, and whether the user is talking or not. First, we discretize the engagement annotation (initially ranging from 0 to 1) on a five items scale (Yannakakis et al., 2018). Then, we rebalance the data using random oversampling (Menardi and Torelli, 2014) to solve the engagement distribution issue in Noxi (70% of NoXi's speaking turns are annotated on the fourth scale, depicting high engagement).

Non-verbal behaviors are annotated frame by frame in the NoXi corpus when our agent estimates an engagement score at each speaking turn. In our work, we consider the ratio of appearance over the turn of each non-verbal behavior. The engagement estimator is composed of five linear layers separated by leaky relu activations (Xu et al., 2015) and dropout layers (Srivastava et al., 2014). Leaky ReLu is an activation function based on ReLu (identity for the positive values) but also has a small slope for negative values, avoiding vanishing gradients and dropout is a regularization technique that temporarily deactivate parts of the network neurons to avoid overfitting. The first hidden layer is 16 units wide and each hidden layer is twice as wide as the previous one. These blocks are separated by batch norm layers. The optimal width and depth of the model were determined using a grid search. The model performs with a mean square error of 1.41.

### 6.2. Topic manager

The dialog manager has a representation of the user's topics preferences to choose when to change the topic and which would be the most relevant topic to maintain the simulated user's engagement. This estimation is computed following the work from Glas and Pelachaud (2018) that updates a value of the estimated engagement $ENG_{u}^{*}\left(t,o_{j}\right)$ for each topic *o*_*j*_ at each turn according to the equation :


(2) $$\begin{array}{l} \begin{array}{lll} ENG_{u}^{*}(t+1,o_{j}) & = & ENG_{u}^{obs}(t,o_{i})SIM(o_{i},o_{j}) \end{array}\\ \begin{array}{l} \;+ENG_{u}^{*}(t,o_{j})(1-SIM(o_{i},o_{j})) \end{array} \end{array}$$

The estimated engagement for the topic *o*_*j*_ is updated based on the engagement observed for the current topic $ENG_{u}^{obs}\left(t,o_{i}\right)$ and the similarity between the topics *o*_*i*_ and *o*_*j*_, *SIM*(*o*_*i*_, *o*_*j*_)∈[0, 1].

### 6.3. Conversational preferences estimator

To estimate users' conversational preferences, we build a two layer LSTM neural network (Hochreiter and Schmidhuber, 1997). A LSTM (Long Short Term Memory) neural network is a recurrent neural network designed to display short term memory. The estimator takes as input: the previous state, the previous action and the obtained reward, and it outputs an estimation of the user conversational preferences. The conversational preferences of the user *t* is in [0, 1]^2^, the first component represents the probability that the user prefers the agent to perform social behavior and the second one the probability that the user prefers the agent to focus on the task. Estimating the conversational preferences as a probability allows the dialog manager to produce subtle behaviors depending on the certainty outputted by the estimator.

**Figure 4:**
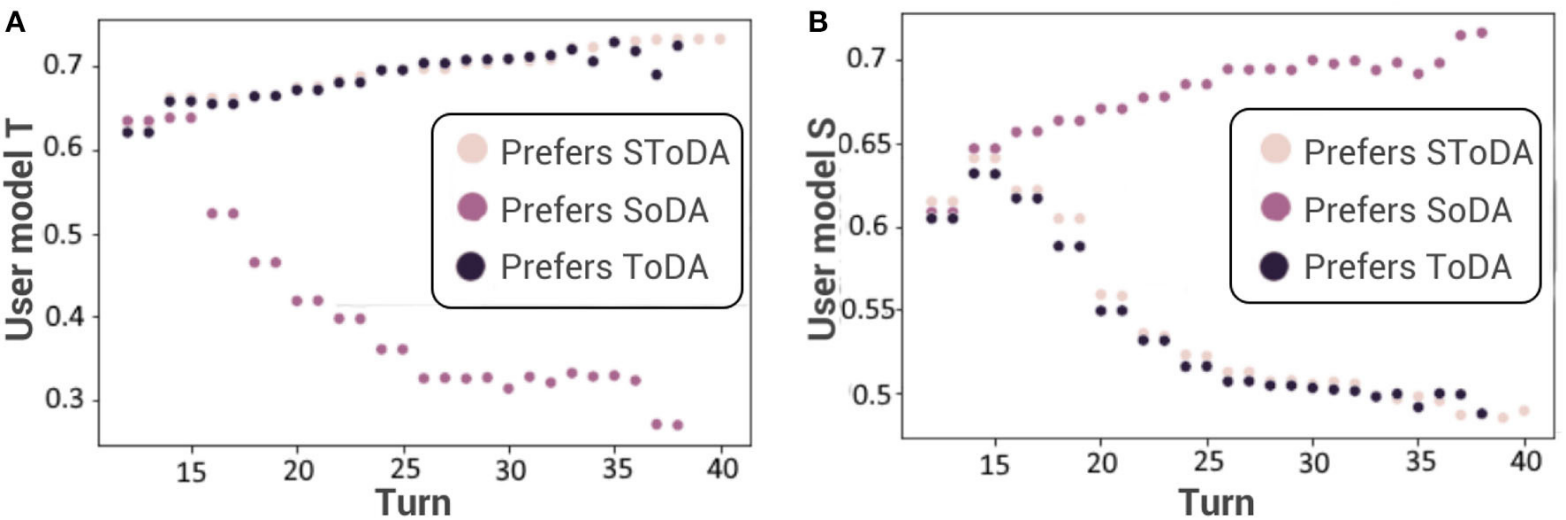
Estimation of the preferred level of task-**(A)** and socially-**(B)** oriented behavior. SoDA, socially-oriented dialog act; ToDA, task-oriented dialog act; SToDA, socially- and task-oriented dialog act.

This approach of estimating conversational preferences detects successfully the conversational preferences of the user during a conversation between our dialog manager and the user simulator with a mean square error of 0.2 (see Figure 4). The user with preferences (TS) is considered as a user with preferences (T). Indeed, focusing on the task is sufficient to reach both goals of the agent: performing the task and keeping the engagement high. Thus, the model chooses to only consider task-driven dialog acts.

### 6.4. Dialog manager

The goal of the dialog manager is to find the optimal dialog policy and to adapt its conversational strategies to the estimated engagement of its user and the estimated conversational preferences of the user. The dialog manager is a Deep Q Neural network (DQN) (Mnih et al., 2013). A DQN is a neural network that takes as input the state of the agent and outputs *Q*-values over the possible actions. the *Q*-values represents the expected reward of each actions. The next action is chosen using a ϵ-greedy policy. The network is optimized to maximize the reward. The state of our dialog manager is:


$$\begin{array}{l} S=\{\text{user's estimated conversational preferences, agent's last }\\ \text{ dialog act, user's last dialog act, mean of the last three }\\ \text{ engagement values, number of turns, estimated topic }\\ \text{ engagement, history of the number of times each }\\ \text{ strategy was used}\}. \end{array}$$

The action space is composed of seven possible dialog acts (task-oriented dialog acts: Inform with a question, Ask a question about the user's knowledge; socially-oriented dialog acts: Joke and Give personal opinion; and neutral dialog acts: Give information, Ask if the user wants to continue or change topic). The reward is crafted to balance task and social rewards (see Table 1). The task reward relates to the transmission of information while the social reward corresponds to the engagement maximization goal and coherent conversational strategies.

**Table 1:** Reward.

**Type**	**Name**	**Value**
Task	Task success	*min*(−200+400*(*n*_inform_−3)/3, 200)
Task	End topic discussed	2 if a topic is finished
Task	Stop	−200 if users walks away
Task	Conv length	−0.001*e*^0.3*t^
Social	Engagement	if detected eng ≥0.5 then 0.01(e^eng^detected^*2^-1)
		else (detected eng - 0.5)
Social	Engagement variation	detected eng–last eng value
Social	Asked question at the wrong time	−0.5
Social	Used the same social strategies twice in a row	−1,000

The dialog manager is a DQN composed of five linear layers separated by leaky relu activations and dropout layers. The first hidden layer is 30 units wide and each hidden layer is twice as wide as the previous one. The three modules (dialog manager, preferences estimator, and engagement estimator) are trained with an Adam optimizer and learning rate of 0.001. The DQN is trained for 500,000 epochs with a buffer of 10,000, a discount factor gamma of 0.8 and an exploration factor epsilon starting at 0.95 and progressively decreasing toward 0.05 in interaction with our simulated user.

## 7. Evaluation

We perform an objective and a subjective evaluation of our model. In this evaluation, our agent takes the role of a museum guide able to present different paintings.

### 7.1. Objective evaluation

#### 7.1.1. Baseline

A baseline, with no adaptation, is developed for comparison with our adaptive model. It is a finite-state machine. The state-space *S* of the machine is: S= { Last agent dialog act, last user dialog act }. The baseline agent has a 40% chance to provide information and a 10% chance to use any other strategy if it has not been used just before.

For each *s*∈*S*, a handcrafted function *P*(*s*):*a*→*p* gives the probability *p* to perform the action *a*. At each speaking turn, given the current state *s*∈*S*, an action is sampled according to this distribution *P*(*s*). The baseline agent does not adapt to users' estimated engagement. The agent has a 40% chance to give information and a 10% chance to use any other strategy if it has not been used just before.

The engagement of the user is then updated and the user performs a dialog act sampled according to its current state (see Section 5).

**Algorithm 1:** Baseline

1: *s*_*agent*_ = (*a*_*agent*_, *a*_*user*_)∈*S*_*agent*_
2: *s*_*user*_ = (*a*_*agent*_, *a*_*user*_, *type*_*user*_, *pref*_*user*_, *eng*_*user*_)∈*S*_*user*_
3: *P*_*agent*_∈*S*_*agent*_ → ℙ(*A*_*agent*_)
4: *P*_*user*_∈*S*_*user*_ → ℙ(*A*_*user*_)
5: while Conversation ≠ Finished **do**
6: *a*_*agent*_←sample[*p*(*s*_*agent*_)] ⊳ Agent performs a dialog act
7: *e*←engagement function(*s*_*user*_) ⊳ The user's engagement is updated
8: *a*_*user*_←sample[*p*(*s*_*user*_)] ⊳ User performs a dialog act
9: *e*←engagement function(*s*_*user*_) ⊳ The user's engagement is updated
10: end **while**

Thus, the agent modeled with this baseline does not adapt to the user with whom it is interacting.

#### 7.1.2. Engagement levels

**Figure 5:**
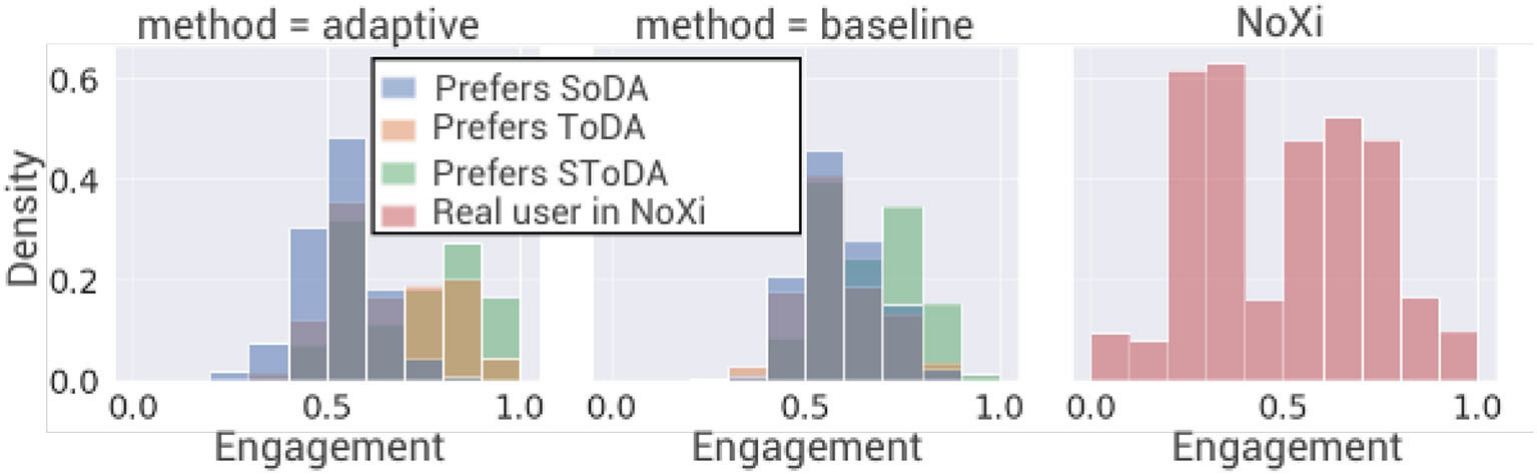
Density of the level of engagement of the simulated user distribution during 1,000 interactions. SoDA, socially-oriented dialog act; ToDA, task-oriented dialog act; SToDA, socially- and task-oriented dialog act.

After training, the average engagement of the simulated user is higher when interacting with our adaptive model (0.63) than when interacting with the baseline (0.6; see Figure 5). A two-way ANOVA is performed to analyze the effect of the simulated user's conversational preferences and of the model adaptability on the engagement of the simulated user. The two-way ANOVA reveals that there is a statistically significant interaction between the effects of the conversational preferences of the simulated user and the adaptability of the model [*F*_(2, 36146)_ = 268.7, *p* ≤ 2*e*−16]. Simple main effect analysis shows that the simulated user's conversational preferences and the model adaptability have each separately a statistically significant effect on the engagement of the simulated user (*p* ≤ 2*e*−16). Tukey's HSD test finds that the mean value of the engagement of the simulated user is significantly higher when the agent is in adaptive condition for users of type (T) (*p* ≤ 2*e*−16, 95% C.I. = [0.05, 0.08]) and (TS) (*p* ≤ 2*e*−16, 95% C.I. = [0.04, 0.06]) and significantly lower for users of type (S). The adaptive model significantly improves the engagement of the simulated user when the latter cares about the task. High engagement for a simulated user who prefers only social behaviors is harder to obtain, since the use of social strategies is not compatible with the task of providing information, which means that the task and the social reward are opposite. However, the model is able to improve the engagement of simulated user who prefers task-oriented behaviors over the baseline.

#### 7.1.3. Dialog act distribution

**Figure 6:**
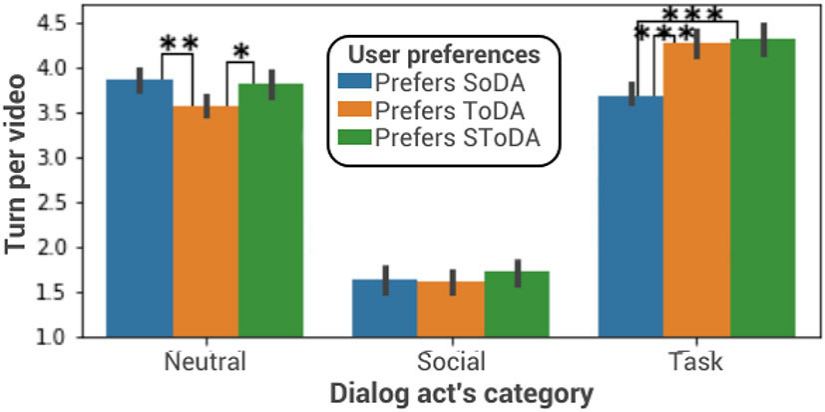
Dialog act distribution. *p ≤ 0.1; **p ≤ 0.01; ***p ≤ 0.001. SoDA, socially-oriented dialog act; ToDA, task-oriented dialog act; SToDA, socially- and task-oriented dialog act.

The trained model favors task-oriented dialog acts when the simulated user prefers to interact with an agent using mainly task-oriented dialog acts (see Figure 6). A one-way ANOVA is performed to compare the effect of the simulated user's conversational preferences on the number of task-oriented dialog acts used by the adaptive model. The one-way ANOVA reveals that there are statistically significant differences in the number of task-oriented dialog acts between at least two groups [F_(2, 970)_ = 25.85, *p* = 1.16e-11]. Tukey's HSD test finds that the mean value of the number of task-oriented dialog acts is significantly different between an agent who interacts with a simulated user who prefers socially oriented dialog acts and an agent who interacts with a simulated user who prefers task-oriented dialog acts (*p* ≤ 2*e*−16, 95% C.I. = [0.4, 0.8]) or with a simulated user who prefers socially- and task-oriented dialog acts (*p* ≤ 2*e*−16, 95% C.I. = [0.4, 0.9]). There is no statistically significant difference between an agent interacting with a simulated user preferring task-oriented dialog acts and an agent interacting with a simulated user who prefers socially- and task-oriented dialog acts (*p* = 0.9). A one-way ANOVA is performed to compare the effect of the simulated user's conversational preferences on the number of socially-oriented dialog acts used by the adaptive model. It reveals that there are no statistically significant differences in the number of socially-oriented dialog acts between at least two groups [*F*_(2, 580)_ = 0.5, *p* = 0.5]. The interaction is also on average 0.4 turn longer when the user prefers task-oriented dialog acts than when it prefers socially-oriented dialog acts. In this case task-oriented users want to learn more about the paintings and therefore the interaction is longer.

#### 7.1.4. Evolution of dialog acts in time

**Figure 7:**
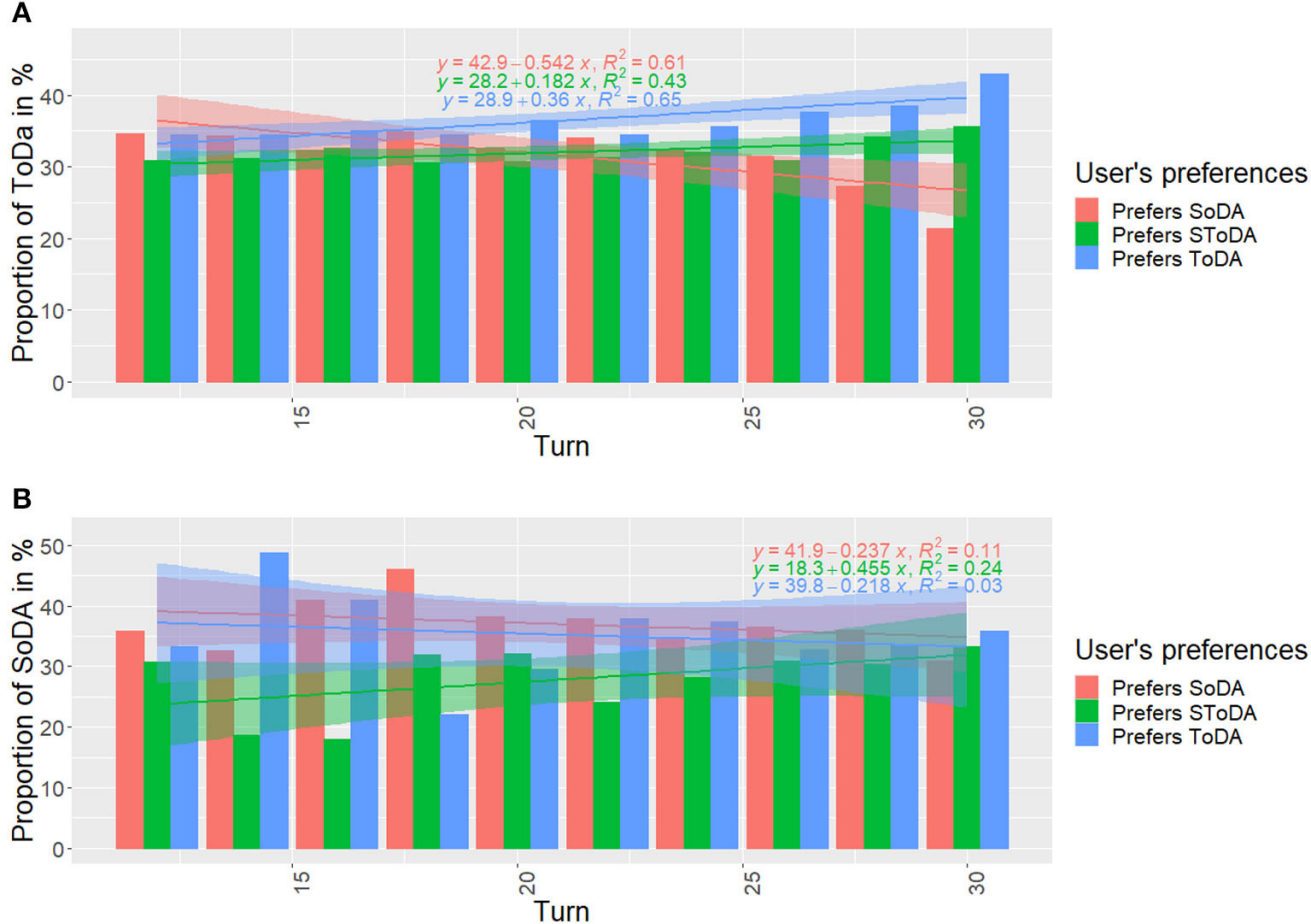
Distribution in time of task-**(A)** and socially-**(B)** oriented dialog acts. SoDA, socially-oriented dialog act; ToDA, task-oriented dialog act; SToDA, socially- and task-oriented dialog act.

As the interaction goes on, the agent gets a better estimation of the type of dialog acts preferred by the current user. Figure 7 shows the proportion of task- (Figure 7A) and socially- (Figure 7B) oriented dialog acts used at each agent turn and for each type of user.

The linear regressions (*prop* = αtime+β) in Figure 7A show that the agent's proportion of task-oriented dialog acts tends to increase over time when interacting with users who prefer task-oriented dialog acts (α = 0.36 and *R*^2^ = 0.65) and users who prefer task- and socially- oriented dialog acts (α = 0.182 and *R*^2^ = 0.43). This proportion tends to decrease when the agent interacts with users who prefer only socially-oriented dialog acts (α = −0.546 and *R*^2^ = 0.61).

The linear regressions (*prop* = αtime+β) in Figure 7B show that the agent's proportion of socially-oriented dialog acts tends to increase over time when interacting with users who prefer task- and socially- oriented dialog acts (α = 0.455 and *R*^2^ = 0.24).

These results show that our agent effectively learned how to adapt its dialog policy over time depending on the conversational preferences of the user it is facing.

### 7.2. User study

We conduct a perceptive study in which human participants are asked to watch and evaluate a video of an agent interacting with a simulated user. We consider different conditions in terms of adaptability mechanisms and simulated user types. To compute the non-verbal behaviors associated with a task-oriented dialog acts and with a socially-oriented dialog acts, we rely on the main dimensions of social attitudes, namely warmth and competence (Fiske et al., 2007). An agent using task-oriented dialog acts aims to convey a set of information to the user and to appear knowledgeable, while an agent using socially-oriented dialog acts has the goal to build and maintain a positive relationship with the user and to appear friendly. We rely on Biancardi et al. (2019b) to map task-oriented dialog acts with behaviors expressing competence and socially-oriented dialog acts with behaviors expressing warmth. We conduct a perceptive study to validate (1) whether participants are able to perceive the adaptability of our conversational agent, (2) whether adaptability influences the perception of the agent's behaviors in terms of warmth and competence, and (3) whether our model improves the perceived quality of the interaction compared to our baseline.

### 7.3. Stimuli

To visualize the conversation, we implement our agent on the Greta platform, which contains an embodied virtual agent capable of communicating verbally and non-verbally with human users and/or other virtual agents (Grimaldi and Pelachaud, 2021). For this study, we associate each dialog act of the agent with a sentence in natural language and a list of non-verbal behaviors. The non-verbal behaviors are generated using the meaning miner module from the Greta platform (Grimaldi and Pelachaud, 2021). This module is based on Image schemas and Ideational units and automatically generates the corresponding non-verbal behaviors. These non verbal behaviors are adapted to the detected user conversational preferences by using the probability that the user expects socially-oriented (here warmth) behaviors [respectively, task-oriented behaviors (here competence)] as the probability that the agent smiles (respectively, cross arms) during its utterance (Biancardi et al., 2017). We use the platform Greta to instantiate these verbal and non-verbal behaviors into sequences of synchronized speech and animations.

Our aim is focused on understanding the adaptability of the virtual agent in term of its choice of dialog acts. To avoid dealing with signal processing and natural language processing in real-time that is a common difficulty in human-agent interaction, we chose to conduct a third-party evaluation study. The study participants are presented with videos of the interaction between our Greta agent and a simulated user. The Greta agent, named Camille, takes the role of a museum guide that can discuss three different topics (i.e., paintings). The simulated user who plays the role of the visitor is not shown directly in the video. To limit the number of variables regarding the user and not to bias participant's perception of the agent, we chose not to display them; but rather show only their engagement level. However, the user's utterances are prompted as subtitles, and the user's engagement level is visualized through a potentiometer. We choose to display the engagement of the user using a potentiometer like in Glas and Pelachaud (2018) as it is a third-party study, and participants are not directly involved in the interaction and cannot see the user. The potentiometer allows participants to have information about user engagement and therefore be better able to judge the interaction^1^.

The scenario of the presented interaction is composed of three phases: an introductory phase, a discussion phase, and a closing phase. The introductory and closing phases are scripted. During the not scripted discussion phase, the agent presents a painting to the user with different possible strategies at each speaking turn. After the end of the interaction, participants completed surveys to measure their perceived quality of the interaction, and their perceived warmth, and competence of the agent.

We identify two between-subject independent variables. One such variable is the adaptability of the agent (Strat-ConvPref), which has two levels. First, the baseline $C_{baseline}^{*}$, where the system does not take into account the engagement of the user and is based on a set of handwritten rules. The second level $C_{model}^{*}$ corresponds to our model, where the agent adapts its conversational strategies to maximize the user's engagement. Our aim is to investigate how participants perceive the adaptation mechanism and whether it improves the perceived quality of the interaction. The second between-subject variable is the conversational preferences of the user (User-ConvPref), which represents the user's conversational preferences and takes 3 values (see Section 5.1.1):

- $C_{*}^{TS}$ Social and task: The simulated user has preferences **(TS)**
- $C_{*}^{S}$ Social: The simulated user has preferences **(S)**
- $C_{*}^{T}$ Task: The simulated user has preferences **(T)**

Thus, we have 6 different conditions: $C_{baseline}^{TS}$, $C_{baseline}^{S}$, $C_{baseline}^{T}$, $C_{model}^{TS}$, $C_{model}^{S}$, $C_{model}^{T}$. We realize 12 videos, two per condition. To balance any possible gender effect, for each condition, we realize one video in which the user is named Alice and one in which the user is named Paul. Two videos of the same condition also discuss different topics to avoid bias introduced by the templates and paintings discussed during the interaction.

### 7.4. Measurement

To measure the simulated user perception of the interaction, participants are asked to rate their agreement from 1 (no agreement) to 5 (high agreement) with a list of six statements adapted from Traum et al. (2012), as well as five statements about their own perception of the interaction including participants' perception of user satisfaction of the interaction; how much the simulated user liked the virtual museum guide; how much the visitor (the simulated user) learned from it; how much the participants would be interested in interacting with the virtual guide; whether the participants feel that the virtual agent adapts to the simulated user; where the participants would place the agent on a scale ranging from computer to a person, and the perceived relationship between the simulated user and the virtual guide from stranger to close friend; whether the user wants to continue the interaction; whether the user wants to know more about the exhibit (see Table 2). The answers are given on a five-step Likert scale.

**Table 2:** Questions to the participants to measure their perception of the agent and of the user.

**Measure**	**Question**
(a) Satisfaction	The user is satisfied with its interaction with Camille
(b) Like	The user liked Camille
(c) Learn	The user has learned something from Camille
(i) Continue visit	Camille made the user want to visit the exposition
(h) Continue	The user would like to talk with Camille again
(e) Adaptation	Camille was responsive to the user's engagement
(d) Interact	I would like to interact with Camille
(*f*_1_) Like-person	I would describe Camille as human-like
(*f*_2_) Like-computer	I would describe Camille as a computer artifact
(*g*_1_) Stranger	I would describe Camille as a stranger to the user.
(*g*_2_) Relationship	I would describe Camille as a close friend to the user

Camille is the agent's name.

To measure how adaptability influences the perception of the agent's behavior, participants are asked to rate how well adjectives corresponding to warmth or competence described the agent (see Table 3; Aragonés et al., 2015).

**Table 3:** Adjectives used to measure agent perceived warmth and competence.

**Warmth adjective**	**Competence adjective**
Kind	Effective
Friendly	Skilled
Pleasant	Competent
Warm	Intelligent

### 7.5. Hypotheses

We hypothesize the following:

- **H**_1_**:** The adaptability type (Strat-Type) delivered by the conversational agent has a main effect on the perceived quality of the interaction. More specifically, interactions when using baseline strategies $C_{baseline}^{*}$ are perceived as worse than interactions when the strategies are adapted $C_{model}^{*}$.
- *H*_2_−*a* (respectively, *H*_2_−*b*): The adaptability type (Strat-Type) interacts with the user conversational preferences (CP) (User-ConvPref) regarding the perceived competence (resp. warmth) of the agent. More specifically, the agent facing a user of conversational preferences (TS) or (T) [respectively, (TS) or (S)] is perceived to be significantly more competent (respectively, warmer) than the agent facing a user with conversational preferences (S) [respectively, (T)] in the adaptability setting $C_{model}^{*}$ only.
- *H*_3_−*a* (respectively, *H*_3_−*b*): The adaptability type (Strat-Type) and the user conversational preferences (User-ConvPref) interact: The perceived competence (respectively, warmth) will be more affected by the type of user (User-ConvPref) in the adaptability setting $C_{model}^{*}$ than in the baseline setting $C_{baseline}^{*}$.

### 7.6. Results and discussion

We collect data from 120 participants (20 per condition) who are recruited using the Prolific crowd-sourcing platform. 20 participants from 2 conditions were removed due to an issue during data collection. We also remove outlier responses. Responses are considered outliers if they are out-of-the-box plots representing the third quartile. In the following, we consider 82 participants for six conditions. Forty-five percent of them were women, 53% were men and 1% other. 17% of them were between 18 and 25 years old, 32% between 26 and 35, 20% between 36 and 45, 26% between 46 and 55, and 4% between 56 and 65. They were all fluent in English and came from various countries, but 58% were from the UK and 20% were from the United States. Ten percent of them were very used to interacting with an artificial agent, 51% moderately, and 38% a little. To compute the warmth and competence score, we compute the Cronbach alpha score on the four adjectives associated with warmth and the four adjectives associated with competence and find good reliability (α = 0.91 and α = 0.84, respectively). For each participant, we compute the warmth and competence score by summing the scores obtained for each adjective. With the same method, the questionnaire associated with the perception of the simulated user of the interaction is very reliable (α = 0.97) and the one associated with the participant's perception of the interaction acceptable reliability (α = 0.67). We sum the score obtained for each question to compute the perception score.

#### 7.6.1. Perception score

On average, the perception of our model is similar to the perception of the baseline, and no significant effects are found. Therefore, our hypothesis *H*_1_ is not verified.

#### 7.6.2. Competence score

**Figure 8:**
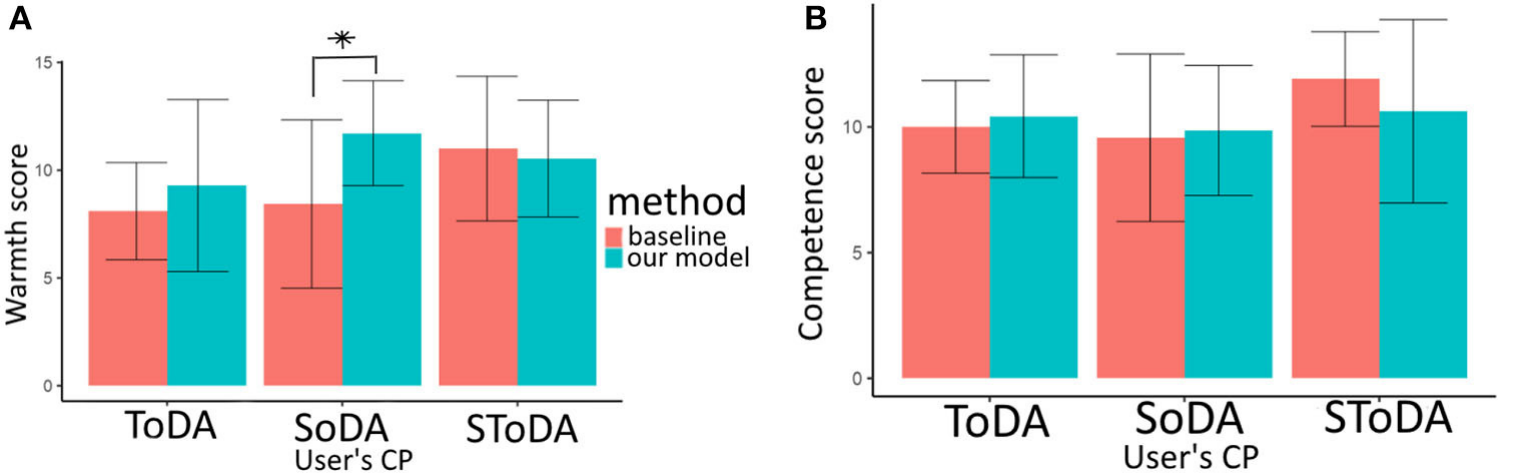
Warmth **(A)** and Competence **(B)** scores. CP, conversational preferences; SoDA, socially-oriented dialog act; ToDA, task-oriented dialog act; SToDA, socially- and task-oriented dialog act. *p ≤ 0.1.

The competence scores are normally distributed (Shapiro's test *p* = 0.016) and their variances are homogeneous (Bartlett's test *p*≥ 0.29). Therefore, we run an ANOVA test. Conditions $C_{model}^{T}$ and $C_{model}^{T}S$ are on average perceived as more competent than conditions $C_{model}^{S}$ (see Figure 8B). This is consistent with *H*_2_−*a*, but no significant effect is found. The difference between the perceived competence of $C_{model}^{T}$ and $C_{model}^{S}$ conditions is larger on average between the conditions of $C_{baseline}^{T}$ and $C_{baseline}^{S}$. This is consistent with *H*_3_−*a*, but no significant effect is found. We can interpret these results as an agent using significantly more task-oriented dialog acts to comply to user's preferences is not perceived significantly more competent than the other agents.

#### 7.6.3. Warmth score

The warmth scores are normally distributed (Shapiro's test *p* = 0.009) and their variances are homogeneous (Bartlett's test *p*≥ 0.46). Therefore, we run an ANOVA test. On average, the perceived warmth of the model is superior when the user expects warmth from the agent (see Figure 8A). This is consistent with hypothesis *H*_2_−*b*, but no significant effect is found. An interaction is found between (Strat-Type) and (User-ConvPref) for the perceived warmth (see Figure 8A). The condition $C_{model}^{S}$ tends to be perceived as warmer than the condition $C_{baseline}^{S}$, *p* = 0.07. Therefore, our results are coherent with *H*_3_−*b*.

#### 7.6.4. Discussion

While the engagement density of the simulated user interacting with our baseline is similar regardless of their conversational preferences, our adaptive agent is able to maximize engagement for two types of user (those who prefer task-oriented dialog acts and those who prefer socially- and task-oriented dialog acts). The results of the objective evaluation also show that our adaptive agent effectively manages to generate significantly different sequences of dialog acts depending on the simulated user's conversational preferences. However, our subjective evaluation shows that human participants did not perceive any significant difference between our adaptive agent and its baseline counterpart, meaning that the sequences of dialog acts generated are not different enough to be perceived by real humans witnessing an interaction. An interesting fact is that our adaptive agent still tends to be perceived warmer than the baseline when it interacts with simulated users who prefer socially-oriented dialog acts, even though they do not generate more socially-oriented dialog acts. This can be explained as the non-verbal behavior of the agent during the interaction is different depending on the simulated user's conversational preferences. Indeed, the instantiation of the dialog acts into non-verbal behaviors differs depending if they are socially or task-oriented (see Section 7.3). The agent may display a smile, cross arms, show beat gesture, etc. to appear either more competent or warmer.

## 8. Conclusion and future work

In this paper, we design a dialog manager that can adapt its conversational strategies to the conversational preferences of the user. This dialog manager is trained using a user simulator. The simulated user is given preferences. Its preferences determine whether it prefers an agent with socially- and/or task-oriented behaviors. The preferences of the users have an impact on the user's next engagement levels and dialog acts. The dialog manager is trained to adapt to user's engagement. To measure the engagement, an engagement estimator is developed that uses as input the user's non-verbal behavior and outputs its estimated engagement level. The dialog manager adapts to the user using a conversational preferences estimator. Finally, the dialog manager itself is a DQN. We observe that adaptation to the user's preferences improves the overall engagement over a non-adaptive baseline. A subjective study in which participants watch a video of an interaction between the agent and a simulated user also shows that the adaptation is perceivable. On average, the agent facing a user preferring socially-oriented behaviors tends to be judged warmer than the baseline. The agent facing a user expecting only task-oriented behavior is found to be more competent than the baseline. The model trained with simulated users display a first correct strategy. However, this policy might be sub-optimal, it can be finetuned with later interactions with real users.To extend this work, a more complicated user simulator can be used to train the model. NoXi novices make some impromptu inform statements (3.97%) in which they add information they already know or change the topic of the conversation. Our simulated users do not have this possibility. The model produces conversations with high quality variability because of natural language repetitions. One possibility thus is to realize more templates and add more possible strategies to the model. A next step to improve the work would also be to test the model with real users and/or test the model with more nuanced simulated users with a non binary combination of users conversational preferences.

## Data availability statement

The raw data supporting the conclusions of this article will be made available by the authors, without undue reservation.

## Author contributions

All authors listed have made a substantial, direct, and intellectual contribution to the work and approved it for publication.

## Funding

This work was partially funded by the ANR-DFG-JST Panorama and ANR-JST-CREST TAPAS (19-JSTS-0001-01) projects.

## Conflict of interest

The authors declare that the research was conducted in the absence of any commercial or financial relationships that could be construed as a potential conflict of interest.

## Publisher's note

All claims expressed in this article are solely those of the authors and do not necessarily represent those of their affiliated organizations, or those of the publisher, the editors and the reviewers. Any product that may be evaluated in this article, or claim that may be made by its manufacturer, is not guaranteed or endorsed by the publisher.

## Footnotes

1 Demo video available at https://youtu.be/6FXrwtlDYPc.
